# Epidemiology and Prognostic Factors of Candidemia in Cancer Patients

**DOI:** 10.1371/journal.pone.0099103

**Published:** 2014-06-05

**Authors:** Hung-Jen Tang, Wei-Lun Liu, Hsin-Lan Lin, Chih-Cheng Lai

**Affiliations:** 1 Department of Medicine, Chi Mei Medical Center, Tainan, Taiwan; 2 Department of Health and Nutrition, Chia Nan University of Pharmacy and Science, Tainan, Taiwan; 3 Department of Intensive Care Medicine, Chi Mei Medical Center, Liouying, Tainan, Taiwan; 4 Department of Nursing, Chi Mei Medical Center, Liouying, Tainan, Taiwan; Columbia University, College of Physicians and Surgeons, United States of America

## Abstract

**Aim:**

The study of candidemia in cancer patients has been limited. This retrospective study aims to investigate the epidemiologic characteristics and prognostic factors of candidemia among cancer patients.

**Materials and Methods:**

From 2009 to 2012, cancer patients with candidemia were identified at a hospital in Taiwan. The medical records of all patients with bloodstream infections due to *Candida* species were retrospectively reviewed.

**Results:**

During the four-year period, a total of 242 episodes of candidemia were identified among cancer patients. Half of these patients were classified as elderly (≥65 years old), and more than 95% of the candidemia episodes were classified as healthcare-associated infections. Among the 242 cancer patients with candidemia, head and neck cancer was the most common, followed by gastrointestinal tract and lung cancer. Additionally, most of the patients had variable underlying conditions, such as the presence of CVC (99%) or prior exposure to broad-spectrum antibiotics (93%) and were receiving an immunosuppressant (86%). Overall, *C. albicans* (n = 132, 54.5%) was the most common pathogen, followed by *C. tropicalis* (n = 52, 21.5%), *C. parapsilosis* (n = 38, 15.7%), and *C. glabrata* (n = 29, 12.0%). Seventeen patients had polycandidal candidemia, and 77 patients had concomitant bacteremia. Approximately one-third of the patients required admission to the intensive care unit (ICU) or mechanical ventilation, and the overall in-hospital mortality was 50.8%. Multivariable analysis showed that the in-hospital mortality was significantly associated with only the non-use of antifungal agents and acute respiratory failure (*P*<.001).

**Conclusions:**

Candidemia can develop in patients with both solid cancer and hematological malignancy, especially for patients with underlying conditions. Overall, the associated morbidity and mortality due to Candidemia remain high. It was also determined that the non-use of antifungal agents and acute respiratory failure conditions were associated with in-hospital mortality.

## Introduction

*Candida* species have become one of the major pathogens that cause nosocomial infections through bloodstream infection (BSI) [1]–[3]. The incidence of candidemia in Taiwan increased markedly from 1980 to the end of the 1990s [3], [5]–[7]. However, the epidemiology of candidemia has changed over time [2]. For example, although *C. albicans* remains the main cause of invasive candidiasis and accounts for more than 50% of all cases, the number and proportion of candidemia cases caused by non-*albicans Candida* species, including *C. tropicalis, C. glabrata,* and *C. parapsilosis*, are increasing in all populations in nosocomial infection settings [3], [4], [6]. Therefore, the understanding of epidemiological findings for candidemia must be updated.

Most cases of candidemia develop inn the patients at risk of receiving invasive procedures/devices or intravascular catheters, broad-spectrum antimicrobial agents, mechanical ventilation, immunosuppressive agents or parenteral nutrition [3]. In addition, malignancy is one of the major risk factors for invasive candidemia among both adult and pediatric patients [8]–[13]. However, studies of candidemia in cancer patients are limited [14]–[22], and most focus on hematological malignancy [14]–[16], [22]. In this study, we retrospectively reviewed the epidemiologic characteristics of candidemia and investigated the prognostic factors of candidemia among cancer patients at a hospital in Taiwan.

## Methods

### Setting and Study Design

This study was retrospectively conducted at the Chi Mei Medical Center, which is a 900-bed hospital in southern Taiwan. The hospital had a total of 136831 admissions and 41440 cancer patient admissions between 2009 and 2012. Cancer patients with candidemia were identified from the Microbiology Laboratory database. The medical records of all patients with BSI due to *Candida* species were retrospectively reviewed, and the following information was collected: age; gender; underlying conditions (history of immunosuppressant drug use, diabetes mellitus, liver cirrhosis, end-stage renal disease, and cancer); risk factors (presence of a central venous catheter (CVC), recent abdominal surgery, and prior use of broad spectrum antibiotics); laboratory data; microbiological findings; and outcomes. The records and information of patients were anonymized and de-identified prior to analysis. Therefore, informed consent was not required and was specifically waived by the Institutional Review Board. An eEthics approval was obtained from the Institution Review Board of Chi Mei Medical Center.

### Candida Isolation

Fungal blood cultures obtained at the hospital from January 2009 to December 2012 were analyzed. BACTEC Myco/F Lytic bottles (Becton Dickinson Microbiology Systems, Sparks, MD) containing 5–10 mL of blood were incubated in the BACTEC 9240 culture system at 35°C. Each patient was included only once at the time of detection of the first bloodstream infection. The identification of *Candida* species was confirmed using the API 20C and Vitek YBC systems (bioMerieux Vitek, St. Louis, MO).

### Definition

The presence of candidemia was defined by at least one set of positive blood cultures for *Candida* species in patients with compatible clinical signs/symptoms of infections. Healthcare-associated infection was defined according to the National Nosocomial Infection Surveillance guideline [23]. The diagnosis and infection focus of candidemia was made based on clinical, bacteriological, and radiological investigations. Catheter-related bloodstream infection (CRBSI) was defined by a positive semi-quantitative tip culture (≧15 colony-forming units), candidemia, and/or high clinical suspicion. Urinary tract infection (UTI) was defined by a positive urine culture with growth of ≥10^5^ CFU/ml and pyuria. If no primary focus could be identified, the candidemia was classified as primary. In-hospital mortality was defined as death due to any cause during hospitalization. Polycandidal candidemia was the assigned classification if at least two *Candida* pathogens grew from the blood samples of any one patient at the same time.

### Statistical Analysis

The continuous variables are expressed as means ± standard deviations. Comparisons between each variable/category were conducted using the chi-square test. A multivariable forward logistic regression model was used to identify risk factors for mortality. All statistical analyses were conducted using the statistical package SPSS for Windows (Version 19.0, SPSS, Chicago, IL, USA), and a *P* value<0.05 was considered statistically significant.

## Results

### Clinical Characteristics

During the four-year period, a total of 242 episodes of candidemia among cancer patients were identified, and the overall incidence of candidemia was 1.77 episode per 1000 admissions (or 5.84 episodes per 1000 cancer patient admissions). The clinical characteristics of the cancer patients with candidemia are summarized in Table 1. The mean age of the patients was 64.3 years, and half were classified as elderly patients ≥65 years old. The Charlson comorbidity index ranged from 2 to 17 (mean 7.8). More than 95% of the candidemia episodes were classified as healthcare-associated infections and identified as CRBSI. Head and neck cancer was the most common, followed by gastrointestinal tract and lung cancer. However, hematological malignancy was only present in 25 (10.7%) patients. In addition to cancer, diabetes mellitus (n = 69, 28.5%) was the most common underlying disease, followed by liver cirrhosis and end-stage renal disease. Moreover, most of the patients had variable underlying conditions, such as the presence of CVC (99%) or prior exposure to broad-spectrum antibiotics (93%) and were receiving an immunosuppressant (86%).

**Table 1:** Clinical features of 242 cancer patients with candidemia.

	No (%) of all patients (n = 242)
Age, years (mean ± SD)	64.3±15.6
Age ≥65 years, no. (%)	127 (52.5)
Male, no. (%)	146 (60.3)
Charlson comorbidity index (mean ± SD)	7.8±2.5
Healthcare-associated infections, no. (%)	240 (99.2)
Source of candidemia	
Central line-related infection	234 (96.7)
Urinary tract infection	5 (2.1)
Primary candidemia	3 (1.2)
Type of cancer	
Head and neck cancer	70 (28.9)
Gastrointestinal tract cancer	64 (26.4)
Lung cancer	32 (13.2)
Liver cancer	24 (9.9)
Genitourinary tract cancer	15 (6.2)
Breast cancer	14 (5.8)
Gynecologic cancer	8 (3.3)
Skin cancer	2 (0.8)
Hematological malignancy	26 (10.7)
*Candida* species	
* C. albicans*	132 (54.5)
* C. tropicalis*	52 (21.5)
* C. parapsilosis*	38 (15.7)
* C. glabrata*	29 (12.0)
* C. guilliermondii*	6 (2.5)
* C. famata*	1 (0.4)
* Candida* species	1 (0.4)
Underlying condition no. (%)	
Diabetes mellitus	69 (28.5)
Liver cirrhosis	26 (10.7)
End-stage renal disease	12 (5.0)
Receiving immunosuppressant	208 (86.0)
Receiving steroid	43 (17.8)
Receiving total parenteral nutrition	32 (13.2)
Presence of central venous catheter	182 (75.2)
Presence of Port-A catheter	224 (92.6)
Presence of double lumen catheter	19 (7.9)
Presence of Foley catheter	176 (72.7)
Recent abdominal surgery	20 (8.3)
Prior exposure to broad-spectrum antibiotic	225 (93.0)
Laboratory findings (mean ± SD)	
White blood cell count (cell/uL)	12592.7±10042.5
Neutrophil cell count (cell/uL)	10389.1±9119.1
Hemoglobin (g/dL)	10.1±1.8
Platelet cell count (cell/uL)	168300±137500
Aspartate transaminase (IU/L)	67.3±86.6
Total-bilirubin (mg/dL)	3.1±4.2
Albumin (g/dL)	2.5±0.5
Urea nitrogen (mg/dL)	35.5±31.9
Serum creatinine (mg/dL)	1.6±1.5
C-reactive protein (mg/L)	112.0±73.3
Procalcitonin	9.4±9.7
Concomitant bacteremia, no. (%)	77 (31.7)
Antifungal agent	
Fluconazole	215 (88.8)
Caspofungin	4 (1.6)
Micafungin	3 (1.2)
No	19 (7.9)
Outcome, no. (%)	
Intensive care unit admission	89 (36.8)
Acute respiratory failure	84 (34.7)
In-hospital mortality	123 (50.8)
Hospital stay, day	38.1±28.6

### Microbiology Findings

**Figure 1:**
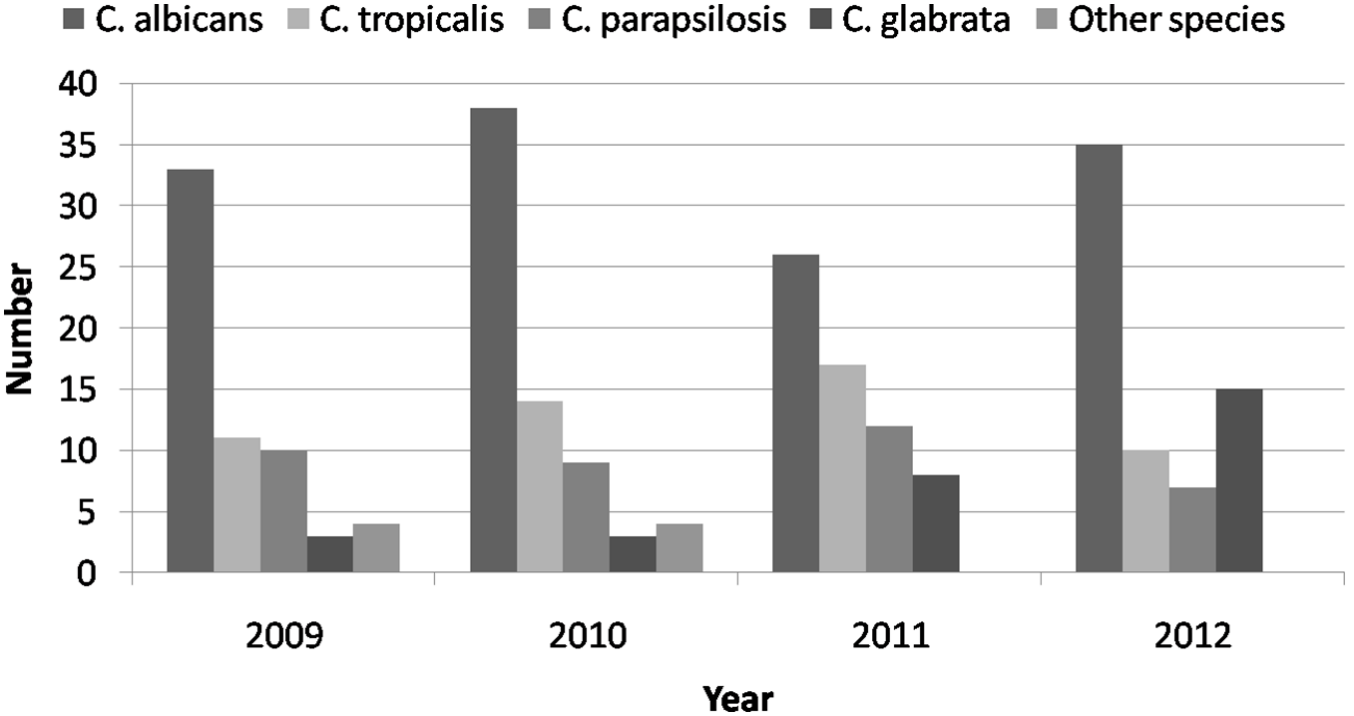
The trend of *Candida* species distribution with year.

**Figure 2:**
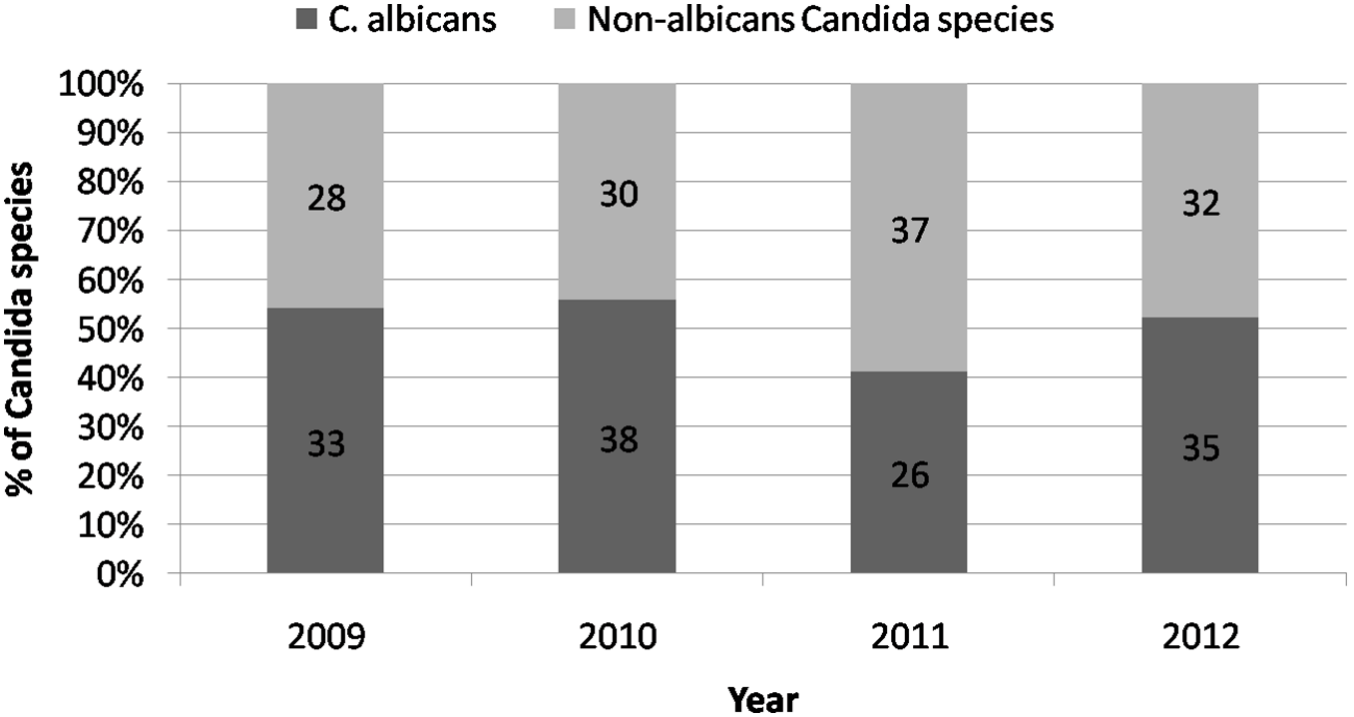
The ratio trends and number of *C. albicans* and non-*albicans Candida* from 2009 to 2012.

Seventeen patients had polycandidal candidemia. Among them, seven had concomitant *C. albicans* and *C. glabrata*, four had *C. albicans* and *C. tropicalis*, three had *C. albicans* and *C. parapsilosis*, two had *C. tropicalis* and *C. parapsilosis*, and one had *C. tropicalis* and *C. glabrata*. Overall, *C. albicans* (n = 132, 54.5%) was the most common pathogen, followed by *C. tropicalis* (n = 52, 21.5%), *C. parapsilosis* (n = 38, 15.7%), and *C. glabrata* (n = 29, 12.0%). The trends of species distribution and the ratio of non-albicans *Candida* by year are shown in Figure 1 and 2. Table 2 summarizes the distribution of *Candida* species among four major cancer categories; however, no significant differences were found among the types of cancer. In addition, 77 patients had concomitant bacteremia. Among these 77 cases, coagulase-negative Staphylococci (n = 34, 44.2%) was the most common bacteria, followed by enterococcus (n = 17, 22.1%), *Staphylococcus aureus* (n = 9, 11.7%), and *E. coli* (n = 8, 10.4%). Moreover, several antibiotic-resistant pathogens were noted, including methicillin-resistant *Staphylococcus aureus* (n = 8), vancomycin-resistant enterococci (n = 6), extend-spectrum β-lactamase (ESBL) *E. coli* (n = 2), and multidrug-resistant *Acinetobacter baumannii* (n = 1).

**Table 2:** The distribution of *Candida* species among four major cancer categories.

	Number (%) of *Candida* species in each type of cancer			
	Head and neckcancer	Gastrointestinal tractcancer	Lungcancer	Hematologicalcancer
*C. albicans*	36 (46.2)	33 (50.8)	18 (51.4)	17 (63.0)
*C. tropicalis*	19 (24.4)	11 (16.9)	5 (14.3)	6 (22.2)
*C. parapsilosis*	13 (16.7)	10 (15.4)	8 (22.9)	2 (7.4)
*C. glabrata*	9 (11.5)	9 (13.8)	3 (8.6)	2 (7.4)
*C. guilliermondii*	1 (1.3)	2 (3.1)	1 (2.9)	0 (0.0)

### Comparison between Patients with Mortality and Survival

Although more than 90% of the patients received a variable antifungal agent, approximately one-third required intensive care unit (ICU) admission or mechanical ventilation, and the overall in-hospital mortality was 50.8%. Table 3 summarizes the comparison between 123 and 119 patients with mortality and survival, respectively. We found that patients with mortality were more likely to be cases with a Charlson comorbidity index >7, infection caused by *C. albicans*, hypoalbuminemia, elevated bilirubin, and no use of antifungal agents than surviving patients. Furthermore, the rates of complications including ICU admission and acute respiratory failure were significantly higher among patients with mortality than survival. Overall, a multivariable analysis showed that the in-hospital mortality was only significantly associated with no use of antifungal agents (odds ratio, 3.498; 95% CI, 1.164–10.508) and acute respiratory failure (odds ratio, 5.119; 95% CI, 2.819–9.293).

**Table 3:** Comparison between patients with mortality and survival.

	No (%) ofpatients withmortality (n = 123)	No (%) ofpatients withsurvival (n = 119)	Univariable			Multivariable		
			Odds ratio	P value	95% CI	Odds ratio	P value	95% CI
Age ≥65 years,no. (%)	60 (48.8)	67 (56.3)	0.739	0.242	0.446–1.226			
Male, no. (%)	71 (57.7)	75 (63.0)	0.801	0.400	0.478–1.342			
Charlsoncomorbidity index >7	82 (66.7)	61 (51.3)	**1.902**	**0.015**	**1.131–3.197**			
Healthcare-associate dinfections, no. (%)	123 (100.0)	117 (98.3)	0.083	0.241	0.960–1.007			
Hematologiccancer	13 (10.6)	13 (10.9)	0.964	0.929	0.427–2.174			
*Candida* species								
* C. albicans*	75 (61.0)	57 (47.9)	**1.700**	**0.042**	**1.020–2.832**			
* C. tropicalis*	25 (20.3)	27 (22.7)	0.869	0.655	0.470–1.606			
* C. parasilosis*	19 (15.4)	19 (16.0)	0.962	0.912	0.481–1.922			
* C. glabrata*	13 (10.6)	16 (13.4)	0.761	0.492	0.349–1.659			
* C. guilliermondii*	2 (1.6)	4 (3.4)	0.475	0.396	0.085–2.644			
* C. famata*	1 (0.8)	0 (0.0)	1.008	1.000	0.992–1.024			
* Candida* species	0 (0.0)	1 (0.8)	0.092	0.492	0.975–1.008			
Underlyingcondition no. (%)								
Diabetes mellitus	34 (27.6)	35 (29.4)	0.917	0.761	0.525–1.602			
Liver cirrhosis	15 (12.2)	11 (9.2)	1.364	0.460	0.599–3.104			
End-stage renal disease	7 (5.7)	5 (4.2)	1.376	0.595	0.424–4.461			
Receiving immunosuppressant drugs	108 (87.8)	100 (84.0)	1.368	0.400	0.660–2.837			
Receiving totalparenteral nutrition	16 (13.0)	16 (13.4)	0.963	0.920	0.457–2.025			
Recentabdominal surgery	10 (8.1)	10 (8.4)	0.965	0.938	0.386–2.409			
Laboratoryfindings (mean ± SD)								
Neutropenia (<500 cell/uL)	5 (4.1)	6 (5.0)	0.798	0.716	0.237–2.688			
Hypoalbuminemia (<2.5 g/dL)	57 (46.3)	31 (26.1)	**2.452**	**0.001**	**1.427–4.213**			
Elevated bilirubin (>2 mg/dL)	28 (22.8)	14 (11.8)	**2.211**	**0.026**	**1.099–4.447**			
Prior exposure tobroad-spectrumantibiotic	117 (95.1)	108 (90.8)	1.986	0.191	0.710–5.555			
Concomitantbacteremia, no. (%)	38 (30.9)	39 (32.8)	0.917	0.754	0.534–1.575			
Antifungal agent								
No	14 (11.8)	5 (4.1)	**2.928**	**0.046**	**1.020–8.405**	**3.498**	**0.026**	**1.164–10.508**
Fluconazole	105 (85.4)	110 (92.4)	0.477	0.086	0.205–1.110			
Micafungin	1 (0.8)	2 (1.7)	0.317	0.323	0.033–3.091			
Caspofungin	3 (2.4)	1 (0.8)	2.950	0.352	0.303–28.766			
Removal of catheter	86 (69.9)	92 (77.3)	0.682	0.194	0.383–1.214			
Complication								
Intensive careunit admission	64 (52.0)	25 (21.0)	**4.079**	**<0.001**	**2.317–7.179**			
Acute respiratory failure	63 (51.2)	21 (17.6)	**4.900**	**<0.001**	**2.718–8.832**	**5.119**	**<0.001**	**2.819–9.293**

## Discussion

This large study investigated the clinical features of candidemia among cancer patients in a single institution and had several significant findings. Although several studies have reported on this issue, most of them focus on hematological malignancy, especially in relation to leukemia [12], [14]–[16], [22]. In contrast to these studies [14]–[16], we found that approximately 90% of the 242 candidemia episodes developed in patients with solid cancers. Moreover, candidemia can occur in any type of solid cancer, especially in the cases of head, neck, and gastrointestinal tract cancer. The differences between our findings and previous studies [14]–[16] may be due to different hospital settings. Our institution is not specific for hematological cancer and may have more patients with solid cancer. However, our findings still indicate that candidemia can develop not only in patients with hematological cancer but also those with solid cancer.

In this study, more than 80% of the cancer patients with candidemia had CVC or a port-A catheter, received an immunosuppressant or had prior exposure to broad-spectrum antibiotics. This finding is consistent with previous studies that found cancer patients with candidemia often have variable underlying conditions, in addition to underlying malignancy [18]–[20]. This result suggests that candidemia could be a result of possible pathogens that cause bloodstream infections in cancer patients, especially in the presence of certain underlying conditions.

Consistent with a previous study [19], we noted that approximately 32% of patients had concomitant bacteremia; this phenomenon is not uncommon for patients with candidemia. Moreover, several antibiotic-resistant pathogens, including coagulase-negative Staphylococci, methicillin-resistant *Staphylococcus aureus*, vancomycin-resistant enterococci, extend-spectrum β-lactamase (ESBL) *E. coli*, and multidrug-resistant *Acinetobacter baumannii* were found to be co-pathogens in cancer patients. This finding reminds us that while treating patients with candidemia, the presence of possible concomitant bacteremia cannot be neglected. In this critical condition, broad-spectrum antibiotics should be administered to cover antibiotic-resistant bacteria.

In this study, the in-hospital mortality due to candidemia remained as high as 50% in cancer patients. This finding is similar to a previous study [19], which found that 30-day crude mortality was 40% in hematology patients and 45% in oncology patients. However, this value is higher than those reported by a study in China [18] and in a Portuguese hospital [20], where the crude mortality rates were 31.7% and 31.9%, respectively. In addition, we found that no use of antifungal agents and acute respiratory failure were the only independent and significant risk factors associated with mortality. This finding is consistent with previous studies [19] and indicates that the appropriate and prompt use of an antifungal agent should be a life-saving measure for cancer patients with candidemia.

We found that *C. albicans* was the most common causative agent of fungemia, accounting for more than half of all episodes of candidemia among cancer patients in our hospital. In addition, *C. tropicalis* (21.5%), *C. parapsilosis* (15.7%), and *C. glabrata* (12.0%) were the most frequently isolated non-albicans *Candida* species. This result differs from a surveillance study of candidemia in cancer patients in China [18], which found that *C. albicans* was the most common species (48.8%), followed by *C. parapsilosis* (24.4%), *C. glabrata* (9.8%), *C. tropicalis* (9.8%), *C. lusitaniae* (4.9%), and *C. famata* (2.3%). Another study [19] in Australia showed that in 133 hematology patients, *C. albicans* was the most common pathogen, followed in frequency by *C. parapsilosis* (n = 27 episodes, 19.5%), *C. krusei* (n  =  23, 16.6%) and *C. glabrata* (n* * =  17, 12.3%), while in 150 oncology patients, the second most common species was *C. glabrata* (n  = 29, 19.3%), followed by *C. parapsilosis* (n* * =  24, 16%). In Portugal [20], among 119 episodes of candidemia in oncology patients, the most frequent species found was *C. albicans* (48.7%), followed by *C. parapsilosis* (20.2%), *C. tropicalis* (8.4%), *C. krusei* (6.7%) and *C. glabrata* (5.0%). However, several studies examining candidemia in Taiwan showed similar findings to those of the present work; *C. tropicalis* was the second most common *Candida* species that caused invasive infections [24]–[27]. Therefore, the epidemiology of candidemia varies among different regions, and each site should regularly conduct surveillance studies to establish the local epidemiology.

There were several limitations to this study. First, we did not determine the *in vitro* antifungal susceptibility profiles of the *Candida* species. Therefore, we cannot evaluate the association between inappropriate antifungal agent and outcome. Second, approximately one-third of the patients treated at our institution had underlying malignancies; therefore, our findings may not be generalized to other hospitals. Third, this study is a retrospective investigation that may suffer from sources of bias, such missing data. However, the impact on this study due to missing data was minimal. Fourth, we used all-cause mortality for outcome analysis and did not evaluate the mortality attributable to candidemia. However, this study attempts to focus on candidemia in oncology patients and still provides useful information regarding this issue.

In conclusion, candidemia can develop in both patients with solid cancer and hematological malignancy, especially among patients with risk factors. Both polycandidal candidemia and concomitant bacteremia are not uncommon in this clinical entity. Overall, candidemia-associated morbidity and mortality remain high. No use of antifungal agents and acute respiratory failure conditions are independently associated with in-hospital mortality. Among cancer patients in Taiwan, *C. albicans* is the most common Candida species, followed by *C. tropicalis*.
